# The Gut-Brain Axis in Neurodegenerative Diseases and Relevance of the Canine Model: A Review

**DOI:** 10.3389/fnagi.2019.00130

**Published:** 2019-06-18

**Authors:** Yoko M. Ambrosini, Dana Borcherding, Anumantha Kanthasamy, Hyun Jung Kim, Auriel A. Willette, Albert Jergens, Karin Allenspach, Jonathan P. Mochel

**Affiliations:** ^1^Department of Biomedical Sciences, College of Veterinary Medicine, Iowa State University, Ames, IA, United States; ^2^Department of Biomedical Engineering, University of Texas at Austin, Austin, TX, United States; ^3^Department of Food Science and Human Nutrition, College of Agriculture and Life Sciences, Iowa State University, Ames, IA, United States; ^4^Department of Veterinary Clinical Sciences, Iowa State University, Ames, IA, United States

**Keywords:** gut-brain axis, neurodegenerative disease, canine, translational, animal models, review

## Abstract

Identifying appropriate animal models is critical in developing translatable *in vitro* and *in vivo* systems for therapeutic drug development and investigating disease pathophysiology. These animal models should have direct biological and translational relevance to the underlying disease they are supposed to mimic. Aging dogs not only naturally develop a cognitive decline in many aspects including learning and memory deficits, but they also exhibit human-like individual variability in the aging process. Neurodegenerative processes that can be observed in both human and canine brains include the progressive accumulation of β-amyloid (Aβ) found as diffuse plaques in the prefrontal cortex (PFC), including the *gyrus proreus (i.e., medial orbital PFC)*, as well as the hippocampus and the cerebral vasculature. Tau pathology, a marker of neurodegeneration and dementia progression, was also found in canine hippocampal synapses. Various epidemiological data show that human patients with neurodegenerative diseases have concurrent intestinal lesions, and histopathological changes in the gastrointestinal (GI) tract occurs decades before neurodegenerative changes. Gut microbiome alterations have also been reported in many neurodegenerative diseases including Alzheimer’s (AD) and Parkinson’s diseases, as well as inflammatory central nervous system (CNS) diseases. Interestingly, the dog gut microbiome more closely resembles human gut microbiome in composition and functional overlap compared to rodent models. This article reviews the physiology of the gut-brain axis (GBA) and its involvement with neurodegenerative diseases in humans. Additionally, we outline the advantages and weaknesses of current *in vitro* and *in vivo* models and discuss future research directions investigating major human neurodegenerative diseases such as AD and Parkinson’s diseases using dogs.

## Introduction

**Figure 1:**
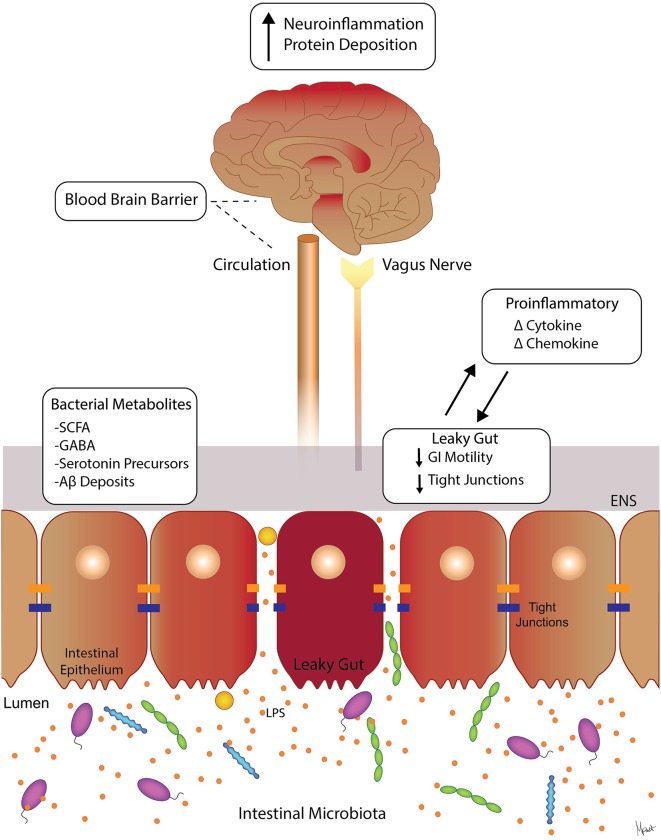
Molecular pathways involved in gut-brain axis (GBA). Suggested signaling pathways and cross-talk between the intestinal microbiota, the intestinal barrier, immune modulators, and neural (brain, vagus, and ENS) systems. The intestinal microbiota can affect the levels of circulating local cytokines, cause “leaky gut” with increase GI permeability, and ultimately affect brain function (Moloney et al., 2014; Carabotti et al., 2015). Intestinal bacterial metabolites such as SCFA, GABA, and serotonin precursors are neuroactive and can affect ENS and the brain (Grider and Piland, 2007; Kimura et al., 2011). Abbreviations: ENS, enteric nervous system; SCFAs, short-chain fatty acids; GABA, γ-aminobutyric acid; LPS, lipopolysaccharide; GI, gastrointestinal.

The gut-brain axis (GBA) is a highly complex interactive network between the gut and the brain, composed of endocrinological, immunological and neural mediators, as summarized in Figure 1 (Rhee et al., 2009). The GBA is largely mediated by the central nervous system (CNS), the enteric nervous system (ENS), and the intestinal microbiota (Grenham et al., 2011). The extrinsic nerves of the gastrointestinal (GI) tract connect the gut to the brain through vagal and spinal afferent fibers, while the brain sends efferent sympathetic and parasympathetic fibers to the GI tract (Grenham et al., 2011; Browning and Travagli, 2014; Foster et al., 2017). The hypothalamic pituitary adrenal (HPA)-axis is known as the main modulator of the physiological stress response but it also modulates alimentary function during digestion (Tsigos and Chrousos, 2002) to facilitate gluconeogenesis. The hypothalamus releases corticotrophin-releasing factor (CRF) and different proteins within this family (e.g., CRF, urocortin 1–3) are also known to affect GI tract function, i.e., intestinal motility (Kihara et al., 2001), permeability (Zheng et al., 2013), and inflammation (Dinan et al., 2006). Specifically, changes in the GI motility induced by urocortin administration were noted in conscious rats, and this study also suggested that the vagal pathway could regulate the central action of urocortin (Kihara et al., 2001). Rats experiencing psychological stress showed decreased level of intestinal epithelial tight junction (TJ) proteins concurrent with increased intestinal permeability in the colon (Zheng et al., 2013). In addition, among patients with irritable bowel syndrome (IBS), the levels of proinflammatory cytokines including interleukin (IL)-6 and IL-8 were elevated as a result of adrenocorticotropic hormone (ACTH) stimulation (i.e., cortisol release; Dinan et al., 2006).

Various studies suggest that intestinal health has a significant impact on neurodegeneration despite the anatomical distance between the gut and the brain (Houser and Tansey, 2017; Zhang et al., 2018). Specifically, dysregulation of GBA cross-talk has been associated with metabolic syndrome (de Lartigue et al., 2011; Grasset et al., 2017) psychiatric disorders such as depression, anxiety, autism, as well as neurodegenerative diseases such as Parkinson’s disease (PD), and Alzheimer’s disease (AD; Sampson et al., 2016; Zhang et al., 2018). In reverse, these neurologic disorders are often times linked to altered intestinal health characterized by changes in the intestinal microbiota composition, which may disrupt the interplay between the gut and the brain (Esteve et al., 2011; O’Mahony et al., 2011). Many studies suggest that the intestinal microbiota contributes not only to modulating the communication and function of the GBA but also to modulating immune response through stimulation of cytokines and chemokines (Moloney et al., 2014). Similarly, the GBA interacts with intestinal cells and the ENS, as well as the CNS through neuroendocrine and metabolic pathways (Carabotti et al., 2015). Furthermore, ENS function can be influenced by the gut microbiota when they locally produce neurotransmitters, including γ-aminobutyric acid (GABA), amino-acid derivatives (e.g., serotonin, melatonin, and histamine) and fatty-acid derivatives (e.g., acetylcholine; Iyer et al., 2004) or biologically active catecholamines (i.e., dopamine and norepinephrine) in the gut lumen (Asano et al., 2012). The ENS is also targeted by bacterial metabolites such as short-chain fatty acids (SCFAs), including acetic acid, butyric acid, and propionic acid, which stimulate the sympathetic nervous system (Grider and Piland, 2007; Kimura et al., 2011), with downstream effects on learning and memory (Vecsey et al., 2007; Stefanko et al., 2009).

## GBA in Neurodegenerative Diseases

**Figure 2:**
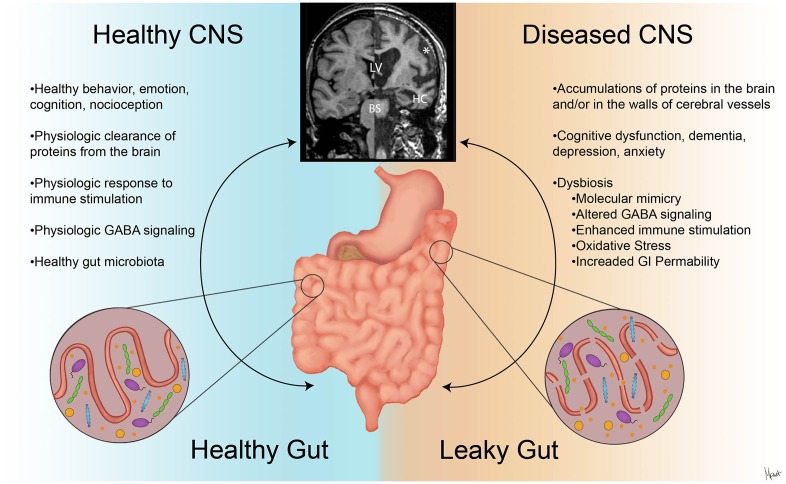
The contrasts of clinical presentations on the GBA in health and neurodegenerative diseases. A stable intestinal microbiota is essential for healthy gut physiology and contributes to appropriate signaling along the GBA, promoting healthy physiologic status as well as central nervous system (CNS) status (*left*). Intestinal dysbiosis can negatively influence gut physiology and lead to abnormal GBA signaling (Friedland, 2015), resulting in accumulation of misfolded amyloid species (Galloway et al., 2007; de Lartigue et al., 2011). This can ultimately alter CNS functions and anatomy (Wu et al., 2017) as shown with magnetic resonance imaging (MRI) volumetric scans (*upper middle*). In the diseased CNS and gut state (*right*), cortical atrophy with the widening of the subarachnoid space (*), enlargement of the lateral ventricles (LV), hippocampus atrophy (HC), and brainstem (BS) volume reduction are seen with clinical and cognitive dysfunction (Johnson et al., 2012; Lee et al., 2015).

Dysfunction of the GBA has been associated with psychiatric disorders including depression and anxiety, as well as neurodegenerative disorders including PD and AD (Sampson et al., 2016; Jiang et al., 2017). The following section will focus on recent findings of GBA involvement in PD and AD and their clinical features, as summarized in Figure 2.

### Alzheimer’s Disease

AD is a progressive neurodegenerative disease characterized by senile plaques consisting of misfolded β-amyloid (Aβ) fibrils and oligomers (Iadanza et al., 2018), as well as hyperphosphorylated tau protein in the various regions of the brain including cerebral cortex, locus coeruleus, and hippocampus (Llorens et al., 2017). Although such protein aggregation in the brain as well as non-neural tissues (i.e., the blood vessels, skin, subcutaneous tissue, and intestine) is a histological feature of AD, such deposition could simply be a consequence of various (epi) genetic alterations triggered by environmental exposures such as sociological, or medical nutritional stress (Lemche, 2018). In fact, synthetic Aß42 peptide aggregation has been reported in *Caenorhabditis elegans* aging models (Patel et al., 2017). Aβ fibrillar accumulation can coincide with clinical signs of cognitive dysfunction (Attems et al., 2005; Herzig et al., 2006; Pistollato et al., 2016), however, it is noteworthy that there is a high degree of variation in the extent of Aβ accumulation among patients with cognitive decline (Monsell et al., 2015). Although almost 100 years have passed since the very first diagnosis of AD, the exact pathogenesis of the disease is still largely unknown (Iadanza et al., 2018). Likewise, no effective therapy for modulation of AD is currently available.

One hypothesis for the involvement of the GBA in the pathophysiology of neurodegenerative diseases is microbial dysbiosis, which occurs as a result of antibiotic exposure (Vangay et al., 2015), dietary changes (Muegge et al., 2011), probiotics (Delzenne et al., 2011), or a variety of other disease conditions (Tilg and Moschen, 2014; Rosenfeld, 2015). Specifically, various studies have shown an association between gut microbiome dysbiosis and the aggregation of Aβ peptides in intestinal epithelial cells (Galloway et al., 2007, 2009) and the CNS (Nam et al., 2017; Lin et al., 2016) after high-fat diet feeding. Different components of the microbiota, such as bacteria, can excrete an immunogenic mixture of functional lipopolysaccharides (LPS), amyloid species, and exudates from their outer membranes into the local intestinal environment (Oli et al., 2012; Schwartz and Boles, 2013). Amyloid species and LPS are usually soluble, although they can polymerize and form insoluble fibrous protein aggregates, leading to stimulation of oxidative stress and cross-seeding of further protein aggregation (Morales et al., 2013; Friedland, 2015; Iadanza et al., 2018). For example, the endotoxin from *Escherichia coli* was shown to enhance the Aβ fibril formation in an *in vitro* model (Asti and Gioglio, 2014). Also, co-incubation of Aβ peptide with LPS was shown to potentiate amyloids fibrillogenesis (Asti and Gioglio, 2014), and systemic injection of LPS in a transgenic AD mouse model resulted in severe amyloid deposition and tau pathology (Aziz et al., 2013; Mitew et al., 2013; Paula-Lima et al., 2013; Saulnier et al., 2013). Moreover, recent studies suggest that the structural overlap between bacterial amyloid proteins to human Aβ could induce *molecular mimicry*, an immune response against the self-antigens stimulated by a foreign antigen sharing structural similarities, and ultimately causing greater inflammatory responses to cerebral Aβ due to altered gut microbiota (Delzenne et al., 2011; Muegge et al., 2011; Rosenfeld, 2015).

Another hypothesis for the pathogenesis of misfolded protein aggregation is the “Prion Concept.” This hypothesis states that many neurodegenerative diseases exhibit accumulation of fibrillary, misfolded proteins similar to the propagation of prionopathies in the CNS (Goedert, 2015). Prionopathy also involves the GBA and the local immune system, where prions accumulate in dendritic cells in the Peyer’s patches and other lymphoid follicles once entering the intestinal epithelium layer (Ano et al., 2009). Interestingly, earlier studies in a senescence-accelerated mouse model identified systemic senile amyloid proteins in Peyer’s patches (Yoshioka et al., 1990). By interacting with dendritic cells, the misfolded protein might be transported to the ENS, and ultimately spread to the CNS compartment (Ano et al., 2009). A significant amount of functional amyloid protein was shown to be generated by certain bacteria, such as *E. coli*, *Bacillus subtilis*, *Salmonella enterica*, *Salmonella typhimurium*, and *Staphylococcus aureus*, and may contribute to the pathology of AD through the accumulation of misfolded Aβ oligomers and fibrils (Hufnagel et al., 2013; Schwartz and Boles, 2013). Some bacterial species, such as *Lactobacillus* spp. and *Bifidobacterium* spp. (both gram-positive bacteria) are known to possess the ability to metabolize glutamate, a well-known primary excitatory neurotransmitter, to produce GABA, a well-known primary inhibitory neurotransmitter (Paula-Lima et al., 2013). These observations suggest that alteration of the gut microbiota can compromise the endogenous production of GABA (Saulnier et al., 2013). In turn, alteration of GABA signaling in the brain has been linked to cognitive impairment, AD, anxiety, and depression (Aziz et al., 2013; Hornig, 2013; Mitew et al., 2013; Paula-Lima et al., 2013). Alternatively, gut bacteria can affect peripheral nerve functions through the production of neuromodulatory metabolites such as short-chain fatty acid (SCFAs; Kimura et al., 2011). SCFAs, i.e., acetic acid, butyric acid, and propionic acid, are produced by bacterial fermentation of dietary fiber in the colon (Kimura et al., 2011). SCFAs can stimulate the sympathetic nervous system to release serotonin, ultimately influencing the CNS cognitive processes such as learning and memory (Grider and Piland, 2007). Catabolism of SCFAs to ketone bodies may also provide an alternative source of ATP to the brain, which could be beneficial given that progressive glucose dysmetabolism has been reported in patients with AD (Sokoloff, 1973). Importantly, lower levels of SCFAs have also been shown to negatively affect immune responses, epithelial cell growth, and possibly affect the function of both the central and peripheral nervous systems (Kimura et al., 2011; Bienenstock et al., 2015).

### Parkinson’s Disease

Patients with PD present with classic motor symptoms, such as asymmetric resting tremor, that are caused by progressive dopaminergic neuronal death in the substantia nigra pars compacta and loss of dopaminergic signaling (Houser and Tansey, 2017). The pathophysiology of neurodegeneration in PD has not been established. However, abundant evidence suggests that neuroinflammation and glial cell activation could play a significant role in PD etiopathogenesis (Rocha et al., 2015). Proinflammatory signaling molecules, including cytokines (i.e., IL-1β, IL-6, and TNF-α; Mogi et al., 1996) or enzymes [i.e., nitric oxide synthase (NOS) and cyclooxygenase-2 (COX-2); Prigione et al., 2009], and oxidative stress are considered major contributing factors to neurodegeneration and cell death in PD.

One of the leading hypotheses for the pathogenesis of PD is the abnormal accumulation of α-synuclein (αSYN; Wong and Krainc, 2017). This protein is present in various cell types in the body, and PD patients show increased expression of αSYN at presynaptic terminals of neurons and neurite projections (Wong and Krainc, 2017). This protein is highly soluble and regulates the presynaptic release of important neurotransmitters such as dopamine (Wong and Krainc, 2017). The αSYN protein is also expressed within the ENS and can be detected in intestinal submucosal neuronal structures from neurologically healthy individuals (Böttner et al., 2012; Shannon et al., 2012; Gold et al., 2013). However, through interactions with environmental factors and other proteins and small molecules (Hasegawa et al., 2002; Breydo et al., 2012), αSYN follows a β-sheet structure formation and loses its physiologic membrane-binding capacity, leading to the aggregation of misfolded proteins forming so-called Lewy neurites and Lewy bodies in dopaminergic neurons of substantia nigra and noradrenergic neurons of the locus coeruleus (Hasegawa et al., 2002). Aggregates of misfolded αSYN proteins decrease mitochondrial complex I activity, thus reducing the physiologic functions of mitochondria, which ultimately leads to oxidative stress in the neuron (Jenner, 2003; Prigione et al., 2009). Individuals with mutations in the αSYN gene *SNCA* or duplication of the wild-type *SNCA* allele are known to develop early-onset, rapidly-progressive PD (Klein and Westenberger, 2012). The spread of αSYN proteins from the ENS to the CNS by transsynaptic cell-to-cell transmission in both sympathetic and parasympathetic nervous systems (Danzer et al., 2012) is the foundation for the “Prion Concept” in PD pathophysiology (Brundin et al., 2016). Multiple studies have demonstrated the presence of αSYN aggregates in intestinal biopsies from clinically normal individuals who would develop PD later in their lives (Braak et al., 2006; Shannon et al., 2012; Hilton et al., 2014). This finding indicates that intestinal αSYN precedes sufficient CNS neurodegeneration to produce motor dysfunctions (Houser and Tansey, 2017). Various clinical GI signs or the characteristic PD ENS pathology often occur before brain functions are actually affected, with constipation being the most common GI complaint in PD (Sakakibara et al., 2003). This is likely due to an increased intestinal transit time both in the small and large intestines of PD patients (Sakakibara et al., 2003). In fact, it has been shown that constipation can be a pre-motor symptom of PD years before the patients present with the clinical signs consistent with CNS degeneration (Gao et al., 2011; Lesser, 2002). In addition, an increased intestinal permeability was shown in PD patients compared to healthy controls (Schwiertz et al., 2018). Other studies suggest that there is an increased risk of developing dementia (Chen et al., 2016) or PD (Lai et al., 2014) in patients with IBS.

Similar to the trend in AD research, the relationships between the intestinal microbiota and PD pathophysiology and their association with disturbed GI motility have been studied extensively and some of the reported differences include a decrease in fecal numbers of *Prevotella* spp. and *Clostridium* spp. in PD patients (Tan et al., 2014; Scheperjans et al., 2015). These intestinal bacteria are a major source of SCFAs, particularly butyrate, folate (vitamin B9), and thiamine (vitamin B1), which are critical for the long-term maintenance of the epithelial barrier function (Tan et al., 2014; Scheperjans et al., 2015). Interestingly, chronic exposure to these SCFAs has been associated with clinical improvement in patients with PD [i.e., decreased dopaminergic degeneration and disruption of blood-brain barrier (BBB)] and clinical symptoms (Luong and Nguyen, 2013; Scheperjans et al., 2015; Liu et al., 2017), possibly due to ketogenesis. Finally, there are a few anecdotal reports suggesting a role for the Tobacco Mosaic virus (TMV; Friedland, 2015) in the pathophysiology of PD, but these preliminary findings need to be consolidated by additional studies on the topic.

## Experimental Approaches to Investigating the GBA

**Figure 3:**
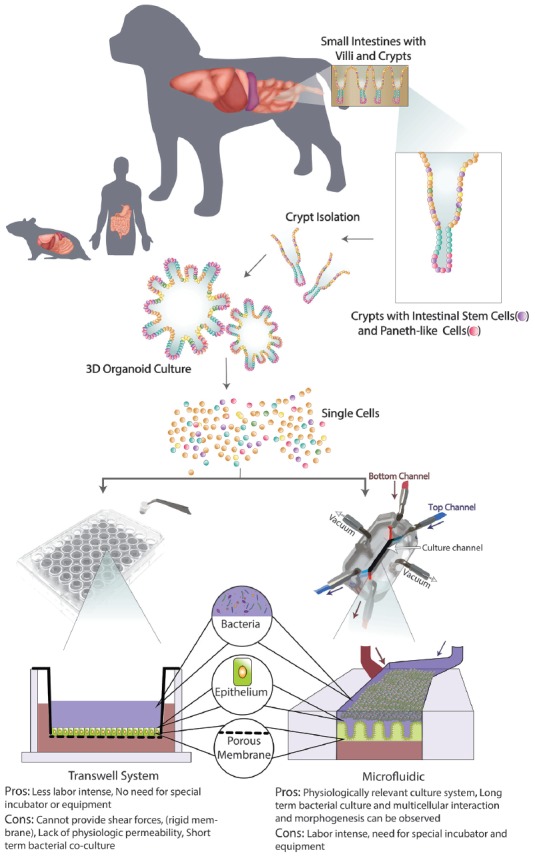
Schematic of organoid 3D culture development and integration into Transwell and Microfluidic systems. First, the intestinal biopsy is obtained *via* endoscopically or surgically, then villi and crypts are isolated with intestinal stem cells (ISCs) and Paneth-like cells. When cultured in an extracellular matrix with appropriate microenvironment factors, long-term culture of 3D canine enteroids/colonoids (ENT/COL) is accomplished. Second, a single cell suspension from such 3D culture system will be integrated with Transwell (left) and microfluidic (right) systems. On the transwell insert, 3D ENT/COL is cultured on top of the porous membrane with culture medium in the apical (blue) side and then submerged in culture medium in the basolateral (red) wells. A schematic of a Gut-on-a-chip (GOAC) microdevice allows a closed system with microtubing. Arrows indicate the direction of the flow of culture medium in the apical (blue) and basolateral (red) microchannels.

**Figure 4:**
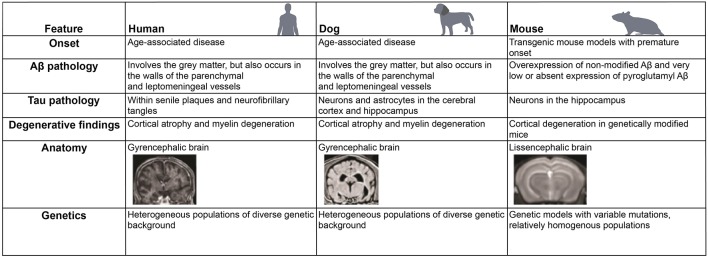
Comparative features of neurodegenerative changes and anatomy in different mammalian species. Similarities and differences in the development of neurodegenerative diseases, such as Alzheimer’s disease (AD), in human, dog, and mouse are listed.

Both static and dynamic *in vitro* models have been utilized to advance the understanding of the role of the GBA in neurodegenerative diseases. In addition, novel primary intestinal stem cell (ISC) culture systems have been utilized to mimic both physiologic and pathophysiologic intestinal conditions *in vitro* contributing to defining gut-cross talk with local environment (Gonzalez et al., 2013; Sato and Clevers, 2013; Chandra et al., 2018). The benefits and disadvantages of two current *in vitro* models are summarized in Figure 3. Importantly, cognitive dysfunction is highly prevalent not only in AD patients but also in approximately one-third of patients with PD (herein referred to as “non-motor symptom” of PD; Chaudhuri et al., 2006). In the “*In Vitro* Models” section and “*In Vivo* Animal Models” section, our main focus will be on AD. However, findings from these *in vivo* models for investigating mechanisms of cognitive impairment would be relevant to PD as well. The similarities and differences of clinical and histological observations in humans, dogs, and rodents are further summarized in Figure 4.

### *In vitro* Models

#### Static Systems

Development of translatable *in vitro* models is critical for elucidating disease pathophysiology and developing effective therapies for neurodegenerative diseases. Currently, only about 7% of investigational compounds tested in phase III clinical trials progress on to the market in neurology (Kola and Landis, 2004). This is worse than the average of 11% success rate of drugs marketed for all disease categories (Kola and Landis, 2004; Adjei et al., 2009). The BBB, a unique interface between the peripheral vascular system and the CNS, is a unique feature of the GBA (Rubin et al., 1991). The critical roles of BBB include supplying nutrients to the CNS, allowing the removal of waste products (such as urea or potassium), and preventing blood-borne pathogens and toxic products from entering into the brain (Alcendor et al., 2012). The BBB consists of TJs between capillary endothelial cells without fenestrations, and therefore allows the BBB to maintain a low level of pinocytosis, which preserves the structural integrity of BBB (Alcendor et al., 2013).

Attempts to develop an *in vitro* model to recapitulate the complexity of the BBB have included brain microvascular endothelial cells and astrocytes in a Transwell culture (Ahmed et al., in press). Leveraging its similarity with conventional 2-dimensional (2D) culture systems and its relative simplicity, the Transwell BBB system has been widely used in a research setting (Rubin et al., 1991). However, the maintenance of TJ function requires the application of the shear forces which traditional static Transwell systems are not able to offer (Santaguida et al., 2006). These critical shortcomings, including lower transepithelial electrical resistance (TEER) and higher endothelial permeability than reported *in vivo*, typically lead to an overestimation of drug permeability across the BBB (Santaguida et al., 2006).

Additionally, current *in vitro* models does not replicate the close physiological cross-talk between pericytes and the capillary endothelium that comprise the neurovascular unit (Jamieson et al., 2017). Successful integration of intraluminal flow for the *in vitro* culture of astrocytes has resulted in more physiological endothelial cell polarity and strengthening of TJs (Cucullo et al., 2011).

Attempts were made to study the GBA using a Transwell culture system as well (Haller et al., 2000). However, this system included only a few components of the GBA, and it is important to note that Caco-2 cells, an immortalized cell line derived from human colorectal adenocarcinoma, are used to model the enteric epithelial cells in this system. Given these collective limitations, as well as the lack of integration of microbiome/ENS in the *in vitro* system, the results derived from these studies may not be readily indicative of translational efficacy.

Importantly, our group recently established canine primary enteroid and colonoid (ENT/COL) culture systems (Kingsbury et al., 2017; Mochel et al., 2017). This is a canine ISC culture system which closely mimics the physiologic structure and function of *in vivo* intestines from both healthy and diseased individuals (Chandra et al., 2018), and allows for the investigation of pathophysiology and treatment effects. Of note, canine cognitive dysfunction (CCD) is a well-studied clinical analog of AD (Kol et al., 2015; Schütt et al., 2015; Hoffman et al., 2018; Wang et al., 2018). Since dogs and humans share an anatomically and physiologically very similar GI tract and harbor a taxonomically and functionally largely overlapping microbiome, the dog provides unique features as a spontaneous model of disease (Coelho et al., 2018; Alessandri et al., 2019). Overall, this canine model may hold promise with its translational relevance for exploration of avenues of novel therapeutics for neurologic disease in the near future (Mochel et al., 2017).

#### Dynamic Model Systems Using Microfluidics

Only recently, a novel *ex vivo* model offering dynamic shear forces to mimic physiologic conditions called organ-on-a-chip (organ-OAC) has emerged (Kimura et al., 2008; Sung et al., 2011). This microfluidic device contains microtubing that allows for continuous flow of media and is comprised of multiple cell culture channels enabling co-culture of different cell types (Kim and Ingber, 2013; Kim et al., 2016a). Specifically, the Gut-on-a-chip (GOAC) models the complex human intestinal anatomy into a two-microchannel device where volumetric flow rate, mechanical deformations, and fluid shear stress can be adjusted to reproduce the *in vivo* physiology of the gut (Kim and Ingber, 2013; Kim et al., 2016a). This biomimetic approach allows for the growth of the villous microarchitecture in Caco-2 cells, while proliferative cells from the intestinal crypt spontaneously migrate toward the villous tip similar to intestinal cells *in vivo* (Kim and Ingber, 2013). Also, differentiation of intestinal epithelial cell lines into four lineage-dependent subtypes (absorptive, mucus-secretory, enteroendocrine, and Paneth) is observed in this microfluidic system and presents a clear advantage over traditional 2D static culture systems (Kim and Ingber, 2013). When Caco-2 cells form 3-dimensional (3D) villi in the GOAC, they typically show enhanced epithelial barrier integrity, increased mucus production, and elevated drug-metabolizing P450 activity with augmented surface area and glucose reuptake, which are all relevant factors for modeling human intestinal physiology (Kim and Ingber, 2013). The 3D microarchitecture and increased mucus production are beneficial to grow live bacteria comprising the human gut microbiota using controlled flow and shear forces mimicking intestinal peristalsis (Kim et al., 2012; Kim et al., 2016a; Shin and Kim, 2018). Steady-state culture conditions inside the microchannels prevent the depletion of nutrients and the overgrowth of microbes (Kim et al., 2012). Critically, overgrowth of bacteria was seen only when manipulation to the shear stress was applied in the GOAC, which emulates the pathophysiological feature of the ileus (Kim et al., 2016a). By leveraging the innovative features of the GOAC, studies on the complex interactions between the host intestinal epithelium and the gut microbiome were also made possible (Kim et al., 2012; Kim et al., 2016b; Shin and Kim, 2018). For example, interactions between the intestinal epithelium, immune cell components, and intestinal bacteria (including non-pathogenic, pathogenic, or probiotic strains) were characterized by adding individual components one-at-a-time in a spatiotemporal manner (Kim et al., 2016a; Shin and Kim, 2018). This approach will enable researchers to evaluate the role of gut microbiome-brain axis in the development and progression of numerous intestinal diseases, such as inflammatory bowel disease (IBD) or colorectal cancer (CRC). Furthermore, the anoxic-oxic interface (AOI) of the oxygen gradient inside a modified GOAC was successfully recreated in a recent report, allowing for the co-culture of strict anaerobic intestinal bacteria and members of the fecal microbiome (Shin et al., 2019). This technology can be used to investigate the cross-talk between the gut microbiome and probiotics on intestinal health.

Recently, a BBB-OAC was established and showed physiological barrier functions (Wang et al., 2017), using ENS and enteroendocrine cells (EEC)-OAC combined to assess the GBA microenvironment (Ahmed et al., in press). Advancement in bioengineering techniques will allow incorporating multiple compartments in one *in vitro* system such as a GBA-OAC (Choe et al., 2017; Ahmed et al., in press; Lee and Sung, 2018). Despite the great promise of the Organ Chip technology, the transfer of cells from a macroscopic environment (e.g., well-plates) to a microfluidic system requires significant revision and optimization of cell culture protocols. Multiple factors differentiate microfluidic from macroscopic cell cultures. Microfluidic systems, for instance, harbor different culture channel surfaces and require fewer media volume as compared with macroscopic cultures (Sung and Shuler, 2009). Despite these limiting factors including the technology being labor-intensive, GOACs are a fast-growing model system that holds greater potential to investigate primary GI diseases. By extension, this system may be able to model GBA microenvironment and brain associations to better understand the role of enteric dysbiosis and neurodegenerative diseases.

### *In vivo* Animal Models

While transgenic rodent models have been utilized to address targeted mechanistic questions relating to neuropathology and altered behavior (Hall and Roberson, 2012, 201), it is important to realize the inherent limitations of these *in vivo* models. Since mouse studies are used in the initial stages of drug discovery, the limitations in this animal model likely contribute to the poor success rate of AD drug discovery over the last 10 years (Kola and Landis, 2004; Adjei et al., 2009). One major limitation in studying the human GBA is a lack of an accurate animal model system that successfully replicates human ENS-microbiome interactions in health and disease. Investigation into the role of GBA with therapeutic interventions may require animal studies with tissues derived from animals that develop naturally occurring disease, including the dog. Since rodent diets differ substantially from that of humans, and diets are an important environmental factor shaping composition of the microbiome, comparing the effect of diet between species that harbor different microbial compositions (and likely functions) is difficult (Flint, 2011; Ravussin et al., 2012). For example, mice preferentially consume grains and cereals which contain relatively low ascorbic acid but have evolved their ability to synthesize this essential cofactor while humans have lost the ability to do so (Perlman, 2016) since they are omnivores. Different cytochrome P450 enzymes exist in mice compared to those in humans, thus each species has unique xenobiotic metabolism pathways that contribute to detoxification in each species (Martignoni et al., 2006; Anderson et al., 2009). These differing means of detoxification may be another reason why toxicology testing in mice has poor translatability to human toxicity (Olson et al., 2000).

Another factor explaining why rodent models do not mirror aspects of human pathophysiology is related to the limited tendency of some of these induced models to develop amyloidosis. As discussed before, AD is histologically characterized by the presence of Aβ aggregates in the walls of cerebral vessels (Attems, 2005; Herzig et al., 2006). Rodent models do not produce human sequence Aβ naturally (Shepherd et al., 2018), which limits their investigative utility as a translational model. Transgenic mouse models overexpressing mutant human amyloid precursor protein (APP) alone, or combined with transgenic presenilin 1 (PS1) and presenilin 2 (PS2), do have secondary Aβ plaque formation in the brain, histologically mimicking AD (Götz et al., 2008). However, these transgenic mouse models naturally have molecular and systemic resistance to Aβ pathology and therefore do not develop the extensive neuronal loss and clinical signs seen in human AD patients (Martin et al., 2011). Lastly, there are fundamental differences in the anatomic folding of the cerebral cortex, with humans having a gyrencephalic brain and rodents having a lissencephalic brain (Sun and Hevner, 2014). A recent meta-analysis study demonstrates that various transgenic mouse models of AD show different characteristics compared to what have observed in the human AD (Hargis and Blalock, 2017). Specifically, the findings from spontaneous AD people were not consistent with those in transgenic AD mouse models, while human studies hold similar findings across different studies (Hargis and Blalock, 2017). The study also found that among the major transgenic AD mouse the findings were not similar to one another (Hargis and Blalock, 2017).

Accumulated data shows that the dog provides a superior model system to transgenic mouse models for investigating the influence of aging in the development and treatment of neurologic disease (Head, 2013). The dog is a more translationally relevant species because of the environmental, genomic, and intestinal physiologic features they share with humans (Cummings et al., 1996). Dogs are an ideal aging model since they show a parallel aging process to humans as evidenced by beagles between 5 and 9 years old showing cognitive dysfunction similar to humans between 40 and 60 years old (Patronek et al., 1997). In addition, brain vs. body size compares favorably between humans and dogs as compared with mice (Roth and Dicke, 2005), which is another advantage of using the dog as a disease animal model for neurologic diseases as canine brains undergo similar stress as humans (Roth and Dicke, 2005). Canine spontaneous disease models also offer additional predictive value for treatment efficacy before transitioning to human clinical trials (Kol et al., 2015; Schütt et al., 2016). Finally, dog genes have adapted to a starch-rich diet during domestication similar to humans, which suggests that studying such adaptations may improve our understanding of human evolution and disease (Axelsson et al., 2013).

### Canine Models as Natural Models for Neurodegenerative Diseases: Similarities and Differences

Many human chronic disorders with a mixed genetic-environmental etiology (e.g., Diabetes Mellitus, IBD, CRC), including AD and PD, have well-studied clinical analogs in dogs (Kol et al., 2015; Schütt et al., 2016; Hoffman et al., 2018). Particularly relevant to AD, aged dogs with CCD spontaneously develop a progressive decline in cognitive function associated with advanced imaging abnormalities and histopathological features similar to AD (Davis and Head, 2014). For example, CCD dogs display progressive AD-like cortical atrophy (Rofina et al., 2006; Pugliese et al., 2010) in areas of the hippocampus that may be accompanied by ventricular enlargement (Su et al., 2005). Further, aged dog brains show other neuropathological and degenerative features similar to AD, including diffuse Aβ plaque deposition (Cummings et al., 1996; Borràs et al., 1999) with cortical amyloid angiopathy (CAA; Ishihara et al., 1991), neuronal loss in temporal regions first affected by AD (Colle et al., 2000), and dysfunction of neurotransmitter systems (Insua et al., 2012). Other neuropathological abnormalities shared between dogs and humans include hyperphosphorylated tau proteins in the brain (Yu et al., 2011; Böttner et al., 2012; Smolek et al., 2016) and increased plasma Aβ_1–42_ levels, one of the biomarkers of AD (Schütt et al., 2015).

In addition to CCD as a model for AD, certain dog breeds are considered spontaneous models for PD. Canine multiple system degeneration (CMSD) is a fatal, inheritable movement disorder first described in Kerry Blue Terriers (deLahunta and Averill, 1976), then in Chinese Crested dogs (O’Brien et al., 2005), and these breeds are considered as natural models for PD. Dogs with CMSD are clinically normal until 3–6 months of age when they first develop the clinical signs of cerebellar ataxia (O’Brien et al., 2005). This progresses to akinesia (i.e., impairment in voluntary movement) and severe postural instability ultimately leading to euthanasia by 1–2 years of age due to a severe decline in quality of life (O’Brien et al., 2005). The histological hallmark of CMSD includes the loss of cerebellar Purkinje cells with degeneration of the olivary nucleus, substantia nigra, and caudate nucleus (deLahunta and Averill, 1976; Montgomery and Storts, 1983), areas of which are relevant to PD etiopathogenesis. Interestingly, the CMSD locus includes a segment that contains *PARK2*, the gene for parkin, and mutations in human *PARK2* is known to cause familial PD, which has clinical and pathological similarities to CMSD (O’Brien et al., 2005).

We acknowledge that there is no perfect animal model for investigating neurodegenerative disorders, and it should be recognized that the canine model also has limitations. For example, it has been recently shown that dogs lack aldehyde oxidases (AOXs) which catalyze the oxidation of aldehydes or N-heterocycles (Terao et al., 2006). This fact has physiological, pharmacological, and toxicological relevance since AOXs represent an important metabolic pathway that oxidizes numerous endogenous and exogenous substrates of biologic importance (Garattini et al., 2003). Also, humans and dogs have different CYP3A isoforms (i.e., canine CYP3A12 is equivalent to human CYP3A4) which impact species-specific differences in permeability, toxicity, and metabolism analysis between *in vitro* and *in vivo* systems (Zhang et al., 2001). A detailed assessment of drug transporters and metabolic enzymes expression *in vitro* is key to establish the predictive performance of these in systems recapitulating *in vivo* drug absorption and metabolism. Also, it is possible that differences in activity and substrate specificity/inhibitors and inducers are observed in the dog; therefore, utilizing *in vitro* systems from multiple different species would allow the supplementation of other *in vitro* systems that do not fully mimic human physiology on their own (Zhang et al., 2001).

## Conclusion

Recent analyses suggest that one of the most expensive therapeutic areas having poor success rate in terms of drug research and discovery (R&D) is neurology (Kola and Landis, 2004). One barrier to achieving lower attrition rates in neurology drug R&D is the lack of utilization of appropriate naturally occurring models of disease, such as CCD as a model for human AD. The dog is a particularly relevant species since it shares multiple epidemiologic features with humans, including similarities in diet and their intestinal microbiomes. Furthermore, CCD dogs can be used as a natural model for both AD, as well as PD, since clinical trials can be performed in dogs to assess the efficacy of novel treatments prior to human trials (i.e., reverse extrapolation). Importantly, since organoids are derived from individuals with different genotypes and environmental exposures, they are a highly relevant model system for *ex vivo* studies, and are of value in “precision medicine.” Integration of organoid culture systems with GOAC technology will maintain patient-specific genetic and epigenetic disease characteristics influencing inter-patient drug screening during the early exploratory R&D phase. We predict that it will be possible to predict the outcome of novel therapeutics prior to human trials by combining data from GOAC models and clinical trials with dogs serving as a model for naturally occurring neurodegenerative diseases.

## Author Contributions

YA and JM conceived the idea for the review. YA searched and reviewed the literature, drafted and revised the manuscript. AW further searched and reviewed the AD and PD literature and revised the manuscript. JM, DB, KA, AJ, AK, and HK reviewed and edited the manuscript. All authors read and approved the final manuscript.

## Conflict of Interest Statement

JM, AJ, KA, and HK are founders of a company, 3D Health Solutions. JM, AJ, and KA are founders of a company, LEAH (Life Engine Animal Health, Inc.) located in Rochester, MN, USA. AK has an equity interest in PK Biosciences Corporation located in Ames, IA, USA. The terms of this arrangement have been reviewed and approved by Iowa State University in accordance with its conflict of interest policies. The remaining authors declare that the research was conducted in the absence of any commercial or financial relationships that could be construed as a potential conflict of interest.
